# Biting Innovations of Mosquito-Based Biomaterials and Medical Devices

**DOI:** 10.3390/ma15134587

**Published:** 2022-06-29

**Authors:** Angela R. Dixon, Isabelle Vondra

**Affiliations:** 1 Department of Biology, College of Arts and Sciences, Case Western Reserve University, Cleveland, OH 44106, USA; 2 Department of Biomedical Engineering, School of Engineering and School of Medicine, Case Western Reserve University, Cleveland, OH 44106, USA; 3 Biomedical Engineering Program, Northern Illinois University, DeKalb, IL 60115, USA; isabelle.vondra@gmail.com

**Keywords:** insect proboscis, insect saliva, bioinspiration, biomimetic microneedles and microprobes, insect-derived anticoagulants, insect-derived polymers, insect eye, nanostructured superhydrophobic coating, insect-based olfactory sensor

## Abstract

Mosquitoes are commonly viewed as pests and deadly predators by humans. Despite this perception, investigations of their survival-based behaviors, select anatomical features, and biological composition have led to the creation of several beneficial technologies for medical applications. In this review, we briefly explore these mosquito-based innovations by discussing how unique characteristics and behaviors of mosquitoes drive the development of select biomaterials and medical devices. Mosquito-inspired microneedles have been fabricated from a variety of materials, including biocompatible metals and polymers, to mimic of the mouthparts that some mosquitoes use to bite a host with minimal injury during blood collection. The salivary components that these mosquitoes use to reduce the clotting of blood extracted during the biting process provide a rich source of anticoagulants that could potentially be integrated into blood-contacting biomaterials or administered in therapeutics to reduce the risk of thrombosis. Mosquito movement, vision, and olfaction are other behaviors that also have the potential for inspiring the development of medically relevant technologies. For instance, viscoelastic proteins that facilitate mosquito movement are being investigated for use in tissue engineering and drug delivery applications. Even the non-wetting nanostructure of a mosquito eye has inspired the creation of a robust superhydrophobic surface coating that shows promise for biomaterial and drug delivery applications. Additionally, biosensors incorporating mosquito olfactory receptors have been built to detect disease-specific volatile organic compounds. Advanced technologies derived from mosquitoes, and insects in general, form a research area that is ripe for exploration and can uncover potential in further dissecting mosquito features for the continued development of novel medical innovations.

## 1. Introduction

Predatory mosquitoes that select humans to feed on have gained notoriety as a bane to human existence [1]. Mosquitoes are known to spread pathogens that cause devastating human diseases, such as Malaria, Yellow fever, and Zika fever [2]. In 2018, mosquito-related diseases were estimated to account for 17% of the global burden of infectious diseases for both morbidity and mortality [3]. Despite their deadly predatory behaviors, the investigation of these and other survival tactics has uncovered how mosquitoes can be beneficial for the development of an assortment of medical technologies.

Evolutionary fine-tuning, especially occurring during associations with humans, has equipped a variety of mosquito species with the physiology and anatomical structures needed to survive and reproduce [4]. Elements isolated or mimicked from the mosquitoes can provide a source of biomaterials and guide the development of medical devices. With procreation being imperative for the survival of mosquito species, female mosquitoes use blood meals as a rich source of proteins and other nutrients for their eggs [4]. Blood-sucking mosquitoes lunge their needle-like proboscis from their mouth deep into the vasculature network within the skin of the host [5]. To counteract the innate processes that prevent blood leakage from the host, mosquitoes apply their saliva at the injury site. Their saliva contains biological agents that inhibit blood clotting and platelet aggregation, allowing uninhibited blood flow and attenuated clotting in lacerated vessels. A host of antihemostatic proteins can be extracted from mosquito saliva [6,7,8,9,10,11], and they are prospective candidates for integration into biomaterial coatings to prevent the thrombosis associated with implantation of medical devices. Additionally, the blood-drawing apparatus of mosquitoes has been replicated in the design of microneedles and other skin-penetrating medical devices to reduce the pain associated with piercing human skin [12,13]. 

Other behaviors that promote the survival of mosquitoes include their ability to move swiftly by using mechanical strength and resilience [14], resist vision obstruction caused by fog in humid environments [15], and use olfaction for feeding and oviposition [16]. These features of mosquitoes have given rise to elastic proteins [17], superhydrophobic materials [18], and olfactory sensors [19], adding to the arsenal of mosquito-enabled tools that can be exploited to enhance human health. 

**Figure 1:**
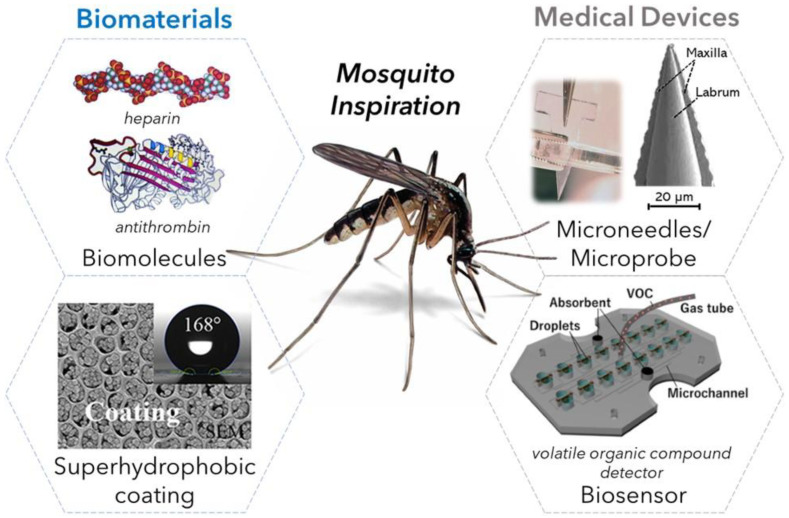
Overview of mosquito-based biomaterials and medical devices. Reprinted/adapted with permission from [13,18,19,20,21].

Similar to many other insects, mosquitoes provide a unique framework, with their internal protein composition and external microstructural features, upon which unique biomaterials and medical devices can be built. Herein, we detail how mosquitoes have inspired unique approaches in the development of biomaterial and medical-device technologies. This brief review discusses the creation of biomaterials and medical devices based on the mimicking of mosquito biting, visual, motor, and olfactory functions. An overview of these mosquito-inspired technologies is shown in Figure 1.

## 2. Mimicking the Mosquito Biting Process

Contrary to widespread belief, not all mosquitoes suck blood. Based on dietary needs, some female mosquitoes opt to drink blood from animals after mating, while male mosquitoes solely drink nectar from plants [4]. The reasoning behind this selective feeding is due to the enrichment of blood with nutrients that are needed for egg production by female mosquitoes [4,22]. Female mosquitoes also drink nectar, but once pregnant, they change their dietary habits to solely blood [23]. Evidence of gender-specific dietary habits that have been engrained evolutionarily is reflected in the design of the mosquito’s mouth [5]. We limit our discussion to the anatomy to the female proboscis, which is designed for piecing the skin and contains blood-sucking apparatuses. 

**Figure 2:**
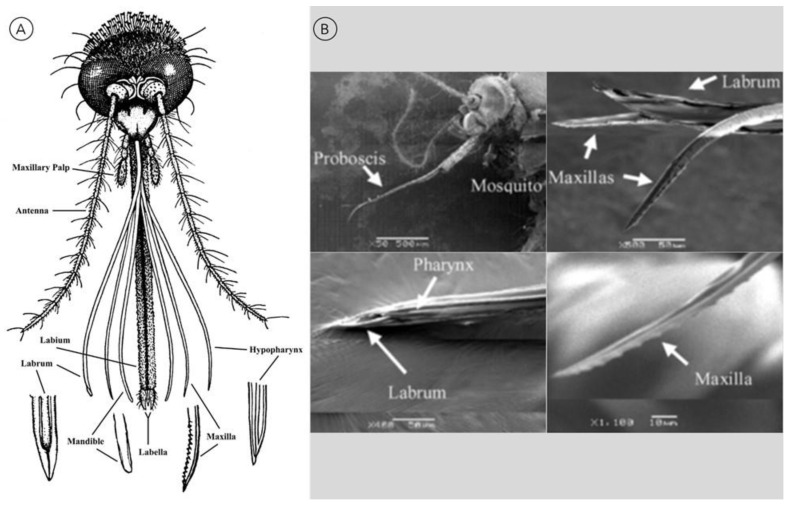
(**A**) Drawing of adult female mosquito mouthparts (labium, stylets, and structure variation at tips). Image obtained with permissions [27]. (**B**) Scanning electron microscopy (SEM) micrographs displaying different mosquito proboscis parts (maxilla, labrum, pharynx). Reprinted/adapted with permissions from [28].

The mosquito proboscis, shown in Figure 2, is a sophisticated needle-like system consisting of many parts that act in unison to pierce through the epidermis of the host [4,24,25,26]. The two major structures of the proboscis are the labium, a retractable and flexible outer sheath; and the fascicle, an internal needle-shaped vessel structure that permits blood flow. A pair of lobes, or labella, that house sensing organs can be found at the tip of the labium [4,25]. Within the fascicle exists six stylets, counting the labrum, hypopharynx, a pair of mandibles, and a pair of maxillae. The labrum is a pointed and hollow tube with “nanosharp” tips that are used to pierce the blood vessels and transport blood. The hypopharynx is another hollow tube, but its purpose is to transport saliva to the blood vessels to either numb the host’s nerves or to create an anti-clotting agent [24,25,26].

As a female mosquito lands on her host animal’s skin, she uses her labium to locate a feeding spot. At a suitable location, the labium rests the labella on the skin and bend backward to help guide and support the fascicle. While it is anchored to the host’s tissues by both the sharp mandibles and saw-like maxillae, the fascicle begins to vibrate, at around 30 Hertz, as it pierces the skin [29]. The hypopharynx releases saliva enriched with biomolecules, including numbing agents, vasodilators, and anticoagulants, at the biting site [5,26]. The flexible labrum plunges into the vessel network in a repeated and aimless manner and uses sensing elements to detect blood. The vibrating labrum lacerates the blood vessel and then draws up the exposed clot-free blood [24,25,26]. 

As described above, the mosquito proboscis contains biochemical and structural elements that enable painless penetration of the host’s skin and facilitate blood feeding. The cooperative vibratory motion of the mosquitoes sharp and jagged mouthparts is credited for permitting painless skin penetration, using three orders of magnitude lower than the insertion force of a commercial needle [25,26]. The saliva released through ducts within the proboscis is replete with several biomolecules that counteract the biting-induced host response [5]. The following subsections discuss how replicating these anatomical, mechanical, and biomolecular features of the mosquito proboscis inspires the development of painless skin-piercing devices and potent anti-coagulant biomaterials. 

### 2.1. Proboscis-Inspired Microneedles

Due to the stealthy biting tactics that mosquitoes employ, most human victims are not aware of the mosquito’s biting action until the blood-drawing process has been completed. Using this covert mechanism of blood extraction, scientists have created painless microneedles to administer drugs and treatments more efficiently via a transdermal route [30,31,32]. Conditions such as aichmophobia, the fear of sharp pointed objects, such as knives, needles, and even the corners of walls, have spurred the investigation of methods to manufacture needles and other sharp-pointed medical devices that are painless and viewed as “less scary” by patients [30]. Refinement of needle design could make the transdermal administration of drugs more efficient and increase patient compliance for mediations requiring routine injections. The anatomical structure and physiological functions of the mouthparts that mosquitoes use to pierce skin and extract blood of their prey have inspired the development of a collection of medical devices that penetrate the skin with ease [24]. 

Many scientists and engineers have redesigned traditional microneedles to effortlessly extract blood from humans by emulating the mosquito-biting process [28,29,30,31,32,33,34], and a collective of researchers affiliated with Kasai University (Osaka, Japan) contributed to a large portion of the studies presented in this section [21,28,29,30,31,33,34,35]. Through close replication of the dimension, shape, and mechanics of different proboscis parts, biomimetic needles can be designed to achieve blood extraction with minimal pain. In this section, we provide an overview and comparison of a representative sample of proboscis-like needles that replicate select features of the mosquito proboscis parts, noting needle size, shape, composition, and mechanical actions. We also discuss how these characteristics are tuned to gain a desired function and performance. Table 1 summarizes the mimicked proboscis part, in addition to shape, size, and material composition details for microneedles discussed. For a description of individual mosquito-inspired microneedle characteristics, along with complementary discussions of skin-penetration mechanics, we direct the reader to review manuscripts by Ramasubramanian et al. and Ramasubramanian and Agarwala [12,24]. Readers can also refer to Lenau et al. for a discussion of biological mechanisms, including skin strain, vibration, and insertion velocity, that enable skin penetration by mosquitoes, as well as the associated experimental methods and skin mimics that are utilized [36].

#### 2.1.1. Microneedle Material Composition

Material composition also plays a crucial role in microneedle performance with respect to mechanics and biocompatibility. Proboscis-like microneedles have been fabricated from a range of materials, including metals, polymers, and other special categories [29,31,35,37,44]. 

Most needles evaluated for this review are composed from the metalloid silicon [28,29,31,35,44,45]. Silicon needles are mechanically desirable given that they require a lower puncture force than conventional metal needles to pierce skin [28]. However, silicon has neither been approved by the FDA nor has it been demonstrated to be biocompatible [29,31]. Silicon microneedles undergo brittle fracture upon insertion, and any released material fragments could be potentially injurious to other parts of the body [29,30,31,35,37,45]. To improve their biocompatibility, some researchers coat silicon needles with a thin layer (2 µm) of parylene, a biocompatible and FDA-approved polymer, but this modification also increases the puncture force required to pierce the skin [29,31]. 

Other metals, including nickel [46], stainless steel [33,38], and titanium [39,40,41], have also been used to form microneedles. Nickel plating can improve microneedle strength but can trigger allergies and sensitivities in the human body [46]. Issues with mechanical strength or human sensitivity have not been reported with the use of stainless-steel microneedles [33]. Titanium is a biocompatible material, but one disadvantage of using titanium needles is that they can buckle under pressure. To mitigate this buckling issue, the diameters and cross-section of the titanium needles can be tuned to increase the sturdiness [39,40,41].

As other notable contenders for biomimetic microneedle fabrication, polymeric materials can offer biocompatibility in various forms, sufficient puncture forces, and resistance to breakage due to flexibility [28]. High-aspect-ratio hollow needles can be readily fabricated in larger volumes with photolithography-based methods by using SU-8, a photosensitive polymer that is also biocompatible and approved by the FDA [37]. Polylactic acid (PLA), which can be broken down in biological environments via hydrolysis to carbon dioxide and water, has also been used to created biodegradable microneedles [28,30]. The puncture force required by a PLA needle to pierce skin is on par with that of a silicon needle, and less than that of a commercial metal needle [28]. In a unique approach, Yoshida et al. investigated a method to repurpose human hair as a microneedle. Hair is composed of keratin, a protein biopolymer [42]. Given that hair is a natural material, it is biocompatible and less likely to cause an immune response if derived from autologous origin. A hair from a Japanese male was subjected to chemical etching with bleach and mechanical drilling to form a hollow pin-shaped microneedle (80 μm external diameter, 20 μm internal diameter, and 2 mm length). The group claimed that the human-hair needle could pierce a chicken leg that simulated human muscle [42]. While this approach is innovative, the research report did not provide details on how the chemical etching process modified the mechanical properties of the hair to permit desirable skin-piercing actions. 

Given that the mosquito proboscis is composed of natural biopolymers, it might be worthwhile to attempt to replicate its precise material composition, geometrical structure, and mechanical properties by using commonly researched natural polymers as building blocks. 

#### 2.1.2. Microneedle Dimensions, Shapes, and Configurations and Associated Performance

The fine dimensions of the mosquito proboscis contribute to its painless piercing of the human dermal skin layer [24,33], which extends 1100 µm deep and is pervaded by capillaries [24], while avoiding pain receptors existing deeper in the skin [12,47]. The entire mosquito proboscis, shown in Figure 2B, spans roughly 2 mm in length and 80 µm in diameter, with the enclosed labrum having a respective outer and inner diameter of around 30 and 20 μm [25]. A close-up view of the proboscis stylet tip (Figure 2B) reveals a straight and tubular labrum, sharpened to a 15° angle at the tip, positioned between a pair of maxillae with harpoon-like jagged edges [33]. Spaced micrometers from the outside of the labrum reside the maxillae that adapt a shape closer to the appearance of a circular arc. Serrated projections line the outside of the tips of the maxillae and decrease in height when approaching the tip, with heights ranging from 2 to 10 μm [48]. The bundle of stylets gradually advances into the skin, with a vibration that generates a sawtooth wave at a frequency of approximately 30 Hz [45]. Biomimetic microneedles that are designed to replicate proboscis dimensions often range between 40 and 100 μm in diameter and between 1000 and 2000 μm in length. The comparatively larger-sized hypodermic needle, whose outer diameter exceeds 100 μm, causes pain upon the penetration into subcutaneous tissue [24].

**Figure 3:**
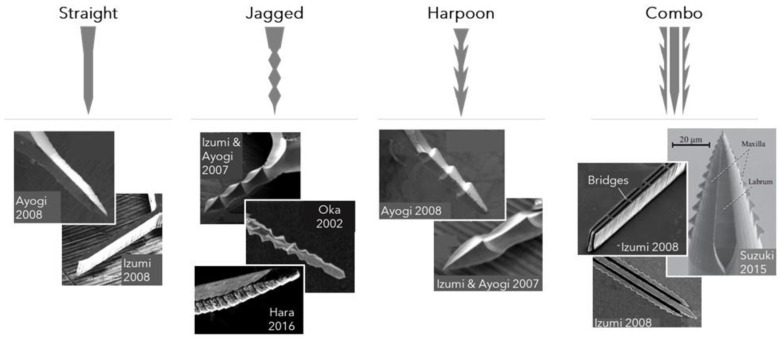
The shape and configuration of some proboscis-inspired needles with straight, jagged, harpoon (complex-jagged), and combination) designs. SEM images reprinted/adapted with permission from [29,30,31,33,35].

Proboscis-inspired needles have been fashioned to conserve the straight or jagged features of the native proboscis that help endow mosquitoes with skin-piercing and blood-sucking functions [21,29,33,34,35,37,38,39]. Beveled hollow needles that mimic the straight and tapered labrum permit ready drawing up of blood [37,38,39,40,41,42]. Solid or hollow needles can include jagged or harpoon-like notches, resembling the serrated edges of the maxillae that saw through and anchor to tissue, facilitating fascicle insertion into the skin [21,29,30,33,34,35]. Composite needle configurations, such as a hollow needle with jagged edges [34,35] or a bundle of straight and jagged needles [21,29], combine multiple shape features to form complex designs that more closely replicate the multipart proboscis structure. Vibrating these anatomically specific needles at a frequency of a few tens of Hz, which mimics the mosquito’s native penetration motion, allows them to pierce skin with minimal damage [28,29,30,34]. The general designs of shaft shape and configurations are shown in Figure 3, along with sample images that display the diversity of shaft designs for proboscis-inspired microneedles.

In straight, jagged, and harpoon shafts, stress generated at the puncture site is concentrated at the needle tip and increases as the needle tip’s angle becomes sharper and the diameter becomes thinner [28,29,30]. Aoyagi et al. performed finite element method (FEM) simulations to determine insertion stresses generated by solid silicon needles, with either a straight, simple jagged, or harpoon-like jagged profile. Simulations involved insertion of the needles into silicon rubber [29], a material commonly used as an artificial skin model [28,29,30,31]. The results revealed that stress concentration occurs solely at the needle tip for straight shapes. However, for jagged shapes, stress is concentrated at the tip areas of both the shaft and its jagged protrusions, and additionally distributed along the side walls. The added regions of stress concentration at many points along the length of jagged shafts facilitate ease of needle insertion. Since harpoon-like shapes have sharper tip angles for their protrusions than simple jagged shapes, stress is more concentrated at the tip areas along the serrated edges of the former. These findings help demonstrate how the harpoon-shaped jutted regions of the mosquito’s maxilla are effective for piercing skin [40]. Another study showed that the application of a 30 Hz vibration reduces puncture forces for all shapes, but to the greatest degree for harpoon-like jagged shapes, underscoring the synergistic effects of shape and penetration motion at needle tips [29].

Compound needle designs have been developed in other research efforts to mimic the labrum and maxillae pair, as a unit, and their synchronized movements. One such composite structure comprises a bundle of three needles, in which a straight tip is sandwiched between two jagged-edged tips [28,29,30]. Various modes of needle motions have been investigated with FEM simulations [30] and skin-penetration models [28] for compound needle designs. In one arrangement in which the central needle advances while the outer jagged needles are stationary, simulation results reveal that stress is confined to a small space around the tip of the needles. The observation is consistent with a scenario where jagged needles anchor to the tissue and allow high puncture stress to be confined between them, permitting the straight needle to advance into the tissue with ease [30]. The “cooperative” alternative motion of the central and outer needles, with a 30 Hz vibration, serves to reduce the skin-penetration force much more effectively than a “non-cooperative” motion that involves the simultaneous insertion of all needles, with or without added vibration [28]. In a separate approach, researchers explored the effects of perpendicularly positioning bridges across the three needles to prevent independent needle movement [29]. In the absence of bridges, the needles can move independently in the lateral direction to generate space for high stress at the puncture site. However, with the addition of 30 Hz vibration, both “linked” and “unlinked” bundles of needles require less force than single-shaft needles to pierce an artificial-skin model [20]. Other tip designs have been explored to streamline the complex mechanics involved with a trio-shaft needle design. A two-part needle with two alternating concave jagged halves reduces the number of needle parts while preserving cooperative motion [34]. A single hollow needle with jagged edges further simplifies the design, but the reciprocating motions that are characteristic of the mosquito proboscis are lost [21,29].

The mosquito proboscis also inspired the design of a needle-cannula system that comprises a cannula covering a serrated biopsy needle that replicated the reciprocating motion of the labrum and maxillae. The design was tested alongside a non-serrated needle, both with and without vibratory motion. During insertion, the non-serrated needle-cannula system moves and deforms tissue, and the deformation and displacement of surrounding tissue is reduced when opposing needle and cannula motions are engaged. The addition of a serrated notch to the needle allows tissue anchoring that further reduces tissue displacement [32]. 

Collectively, these results suggest that the shapes and configurations of needle tips can be tuned to achieve different levels of insertion ease. Moreover, complex designs that more closely mimic the multi-part and multi-shaped elements and physiologically relevant motions of the native proboscis yield the closest replication of mosquito biting actions. 

### 2.2. Proboscis-Inspired Microelectrode Guide 

Microstimulation can be used to stimulate the brain or lesion neural tissue with local application of an excess amount of current through a microelectrode, a technique used to treat a variety of neurological disorders, including Parkinson’s disease and epilepsy [49,50]. The success of this technique can be hampered by the buckling of the microelectrode, which can be severe enough to break the blood–brain barrier and trigger a foreign body response. Fibrous tissue will then encapsulate the electrodes, resulting in eventual rejection through both electronic and physical isolation from the neural system [13]. Researchers have looked to the replication of fascicle anatomy to optimize the design of microelectrodes used in microstimulation-brain-data recording to minimize injury and other adverse biological events [13]. 

**Figure 4:**
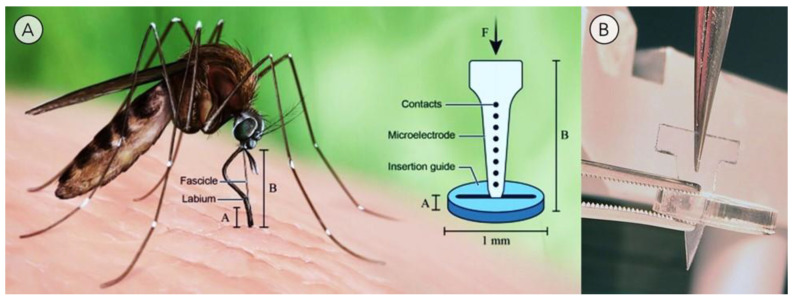
(**A**) Mosquitos use their labium as an insertion guide to prevent buckling of the fascicle as it is inserted into the skin. This cooperative function of the mosquito labium and fascicle inspired the design of a guide that reduces the buckling of a microprobe during its insertion into the brain [13]. (**B**) Photograph of a dummy polyethylene microelectrode probe inserted through a guide. Courtesy of Dr. Capadona Lab.

Shofstal et al. developed a polymethyl methacrylate (PMMA) guide tool to allow for the ease of probe insertion into the brain by mimicking how the labium guides the fascicle into the skin (Figure 4A) [13]. This mosquito-inspired guide tool (Figure 4B) prevents buckling upon the insertion of microelectrodes into the brain. Specifically, when a dummy microprobe was tested in agar gel that served as a model of the rat brain, the rate of successful insertion increased from 23% to 92% and the rate of buckling decreased from 85% to 19%, compared to conditions without a guide. During in-vivo implantations in the rat motor cortex, the guide increased the rate of successful insertion from 37.5% to 100% for microprobes controlled with an automated insertion system [13].

### 2.3. Mosquito-Derived Polymers with Anticoagulant Properties

When the blood vessel is impaled by the mosquito’s proboscis, exposed tissue factor and collagen can lead to platelet aggregation, triggering extrinsic clotting events [51]. The host coagulation response, resulting from injury to the vascular endothelium, can also occur when blood encounters implanted medical devices [52,53,54]. Since an implantable device is not covered by a layer of endothelial cells, plasma proteins (including fibrinogen and von Willebrand factor (vWF)) are deposited onto the surface and interact with other hemostatic components in the bloodstream [47]. In this manner, the intrinsic pathway of the coagulation cascade is triggered, allowing clotting events to occur even when no laceration is present. However, instead of the fibrin clots plugging a hole in the wall of an injured vessel, a provisional fibrin matrix will form around the surface of the biomaterial. This phenomenon is very closely linked with the body’s immune response to the biomaterial. The provisional matrix is a source of chemoattractants and other biochemical signals that mediate the foreign body immune response, which can progress to fibrous-capsule formation around an implanted device [55]. This action not only increases the probability of device failure, but it could also result in decreased or inhibited blood flow to vessels and arteries, leading to cellular atrophy [55]. Another concern that arises with the use of some blood-contacting biomedical devices is the increased risk that thrombosis may occur and lead to adverse conditions and higher patient morbidity and mortality [56]. In contrast to medical devices devoid of anticoagulant agents, the mosquito proboscis, upon insertion into the body, excretes salvia enriched with proteins that inhibit clotting events that would otherwise occur as a response to the endothelial damage [5,6,7,8,9,10,11,57]. Due to their antihemostatic properties, these mosquito salivary proteins could serve as prospective candidates for integration into blood-contacting biomaterials to prevent clot formation and device rejection.

Existing methods to address the detrimental events associated with blood–biomaterial interaction include the use of polymeric and non-polymeric coatings that either elute or incorporate grafted anticoagulant agents [58,59]. One of the most common therapeutic agents that is administered intravenously to prevent clot formation is heparin [60]. Heparin can also be attached to polymer films and used to coat medical implants to prevent thrombus formation [58,61]. Despite heparin’s potent anticoagulant activity, patients using heparin as a pharmacological therapy can possibly develop heparin-induced thrombocytopenia (HIT), a paradoxical prothrombotic immune response that increases the risk of clotting [60]. Heparin biomaterial coatings are also suspected to contribute to HIT, but more research investigation is needed to support this idea [48]. Natural polymers found in mosquitoes can potentially inspire the development of alternative sources of polymer-based antithrombotics that can be incorporated into biomaterial coatings and pharmacological anticoagulant drug design.

**Figure 5:**
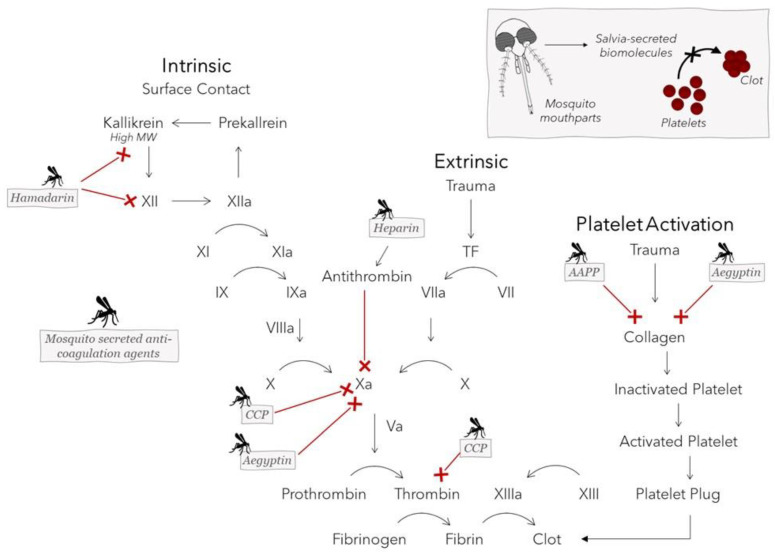
Anopheline antiplatelet protein (AAPP), CPP protein, Aegyptin, hamadarin, and heparin are biomolecules secreted in the saliva of different mosquito species that interfere with blood-clotting events to aid in the ingestion of a blood meal. Select targets of the coagulation cascade and collagen-stimulated platelet activation pathway are inhibited by these mosquito salivary proteins.

Anopheline antiplatelet protein (AAPP), CPP protein (derived from *Culex pipiens pallens*), Aegyptin, hamadarin, and even heparin itself are all biological molecules contained in the saliva of different mosquito species that aid in the ingestion of a blood meal by interfering with the host clotting cascade or platelet aggregation [5,6,7,8,9,10,11,57]. These biomolecules can either directly bind to collagen, preventing its adhesion to platelets, or can inhibit one or more factors in the coagulation cascade. AAPP directly binds to collagen to block platelet adhesion to collagen via glycoprotein VI (GPVI) and integrin 21 [10]. Due to its sequence and functional similarities to AAPP, Aegyptin prevents the interaction of collagen with GPVI and integrin a2b1 [10]. Aegyptin additionally blocks binding of collagen to vWF, a large multimeric glycoprotein that tethers platelets at high shear rates and inhibits factor Xa in the presence of calcium [7,57,62]. By binding to and activating antithrombin, heparin is known to inhibit thrombin and factor Xa, which have an essential role in the final stages of blood clotting leading up to fibrin clot formation [63]. CPP protein appears to have a repressive effect on the enzymatic activity of the proteinases, factor Xa and thrombin, through either direct or indirect inhibitory mechanisms. Although it was observed to inhibit platelet aggregation, the mechanisms that CPP uses to limit the coagulation cascade are not clearly understood [8]. Finally, hamadarin executes its action though interference with the plasma contact system by binding to factor XII and high-molecular-weight kinnogen. Both of these latter factors, in turn, play a role in the formation of kallikrein, which further catalyzes the conversion of factor XII into factor XIIa, allowing the contact system’s sequence to be carried out [11]. Figure 5 shows the effects that these mosquito salivary proteins exert on various targets of the coagulation cascade. Salvia-derived mosquito biomolecules are promising candidates not only for use as therapeutic anticoagulant drugs to treat dangerous blood clots, such as those that cause myocardial infarction and transient ischemic attack [64], but also for incorporation into blood-contacting biomaterials to minimize the risk of device failure resulting from platelet aggregation. A summary of antihemostatic agents produced by different mosquito species and the molecular mechanisms used to counteract host hemostasis to enable feeding can be found in Table 2.

## 3. Mimicking Mosquito Visual, Motor, and Olfactory Functions

**Figure 6:**
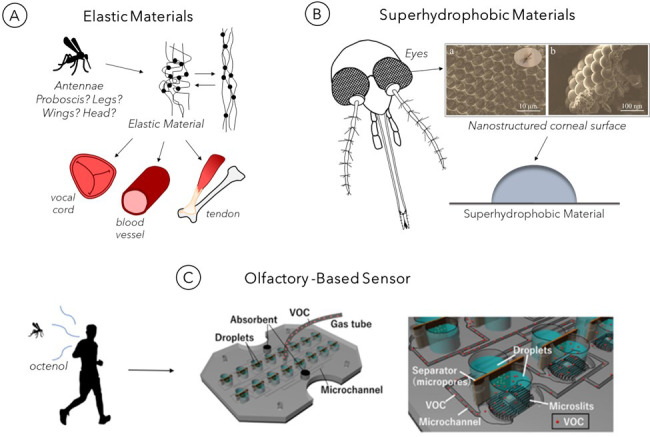
Mosquito-based technological developments based on behaviors other than feeding. (**A**) Mosquito-derived resilin protein, which endows mosquitos with movement abilities, is a multi-responsive biopolymer that can used for tissue-engineering applications, such as vocal-cord, blood-vessel, and tendon repair. (**B**) The nanostructure of the mosquito eye that allows mosquitos to see in humid environments is replicated in the design of superhydrophobic material coatings. Inset displays SEM images of (a) microhemispherical ommatidia that are covered with (b) nanonipples. SEM images reprinted/adapted with permission from [18]. (**C**) The olfactory receptors of mosquitoes can be incorporated into a chip to permit detection of odors emitted by humans with high specificity and sensitivity. This mosquito-inspired volatile organic compound sensor has the potential for detecting human diseases based on volatile biomarkers. Reprinted/adapted with permissions from [19].

Although mosquitoes are well-known for their blood feeding, they exhibit several other adaptive features for survival that can also be applied innovatively. The following section showcases select biomaterials and devices that are inspired by mosquito movement, vision, and olfaction (summarized in Figure 6).

### 3.1. Mosquito-Derived Elastic Resilin-like Proteins

Due to its energy storage and elastic properties, resilin was discovered to be integral to an insect’s mechanical abilities, including flying [65], jumping [66], and vocalization [67]. In *Drosophila melanogaster*, a distant relative of mosquitoes (insects share the same taxonomic order (Diptera) [68], resilin has been detected throughout various regions of the body, including the proboscis, legs and leg joints, wing articulations, and head [69], and it is likely that closely related insects such as mosquitoes share a similar distribution pattern. Resilin in the mosquito has been specifically identified in the spermatheca, a female reproductive organ [70]; and the antenna, a sensory organ [71]. Variability in the material composition of the antennae for different mosquito species and its effect on the antennal resonant frequency has been observed in males [71]. This feature likely evolved for mate selectivity [71], as mosquitoes use wing movement to generate a sound frequency that both males and females can modulate to match with each other during mate selection [72]. Non-biting midges belong to the same Diptera order and Nematocera suborder as mosquitoes [73]. The non-biting midges exhibit sexual dimorphism in antennal resilin composition that influences resonant frequency. This compositional characteristic is suspected to exist for mosquitoes, whose structural sexual dimorphism is apparent [74].

Resilin and resilin-like proteins (RLPs) are actively being used and investigated for many tissue engineering applications [17,75] to create elastic tissue scaffolds for the repair of damaged vasculature [76], tendons [77], and vocal folds [78,79]; and in drug delivery for the development of nanoparticles [80]. Resilin synthesized from genes encoding RLPs identified in *Anopheles gambiae* mosquitoes and *Drosophila melanogaster* fruit flies [81] could be useful for biomedical applications. Recently, resilin-like sequences, An16 and RZ10-RGD, have been derived from genes of *An. gambiae* mosquitoes [81]. 

An16, consisting of sixteen repeats of GAPAQTPSSQY, can be purified from a set of proteins that are encoded by the gene BX619161 expressed in the *An. gambiae* species [81]. An16 possesses molecular flexibility and responds to multiple stimuli, including pH, hydration, and temperature, and these traits are characteristic of RLPs [82]. At basic or physiological pH levels, An16 peptides orient to form protein particle structures that contain a positively charged core and negatively charged surface [81]. Additionally, An16 becomes more resilient and elastic with increasing levels of hydration. In contrast to elastin-like-proteins, An16 exhibits both an upper critical solution temperature (UCST) and lower critical solution temperature (LCST), above and below which miscibility respectively occurs [82,83]. Due to its pH, humidity, and thermal responsiveness features, An16 holds promise as a candidate for biomaterial applications, such as controlled-release drug delivery systems, biosensors, and tissue engineering scaffolds [83]. 

RZ10-RGD, another RLP with tunable glass transition temperature and elasticity, consists of ten repeats of a consensus resilin-like sequence. RZ10-RGD exhibits similar compressive strength to human articular cartilage [84], a relatively elastic tissue that has limited ability for intrinsic healing and mechanical properties that present challenges for tissue repair [85]. A hydrogel created by crosslinking RZ10-RGD with 3, 3′-dithiobis (sulfosuccinimidyl propionate), a redox-responsive crosslinker, was investigated for potential effectiveness in targeted drug delivery and tissue engineering [86]. The RZ10-RGD hydrogels possessed a well-connected network structure, with an average pore size of ~10 µm. The high cytocompatibility of RZ10-RGD hydrogels was demonstrated by examining the viability of NIH/3T3 fibroblasts cultured on the hydrogel. Additionally, the hydrogels displayed a rapid degradation rate in reducing environments [86], such as those maintained by cells and tissues to preserve electrochemical gradients and guard against oxidative stress for survival [87]. Based on these observations of biocompatibility, RZ10-RGD hydrogels are ideal for tissue engineering and drug delivery applications.

### 3.2. Mosquito Eye-Inspired Superhydrophobic Coating

The micro- and nanostructure of mosquito eyes gives rise to a superhydrophobic trait that helps them see clearly while navigating and enduring the damp and humid environments required for breeding [15]. Researchers have created superhydrophobic surfaces that mimic mosquito corneal surfaces [18], and these materials may have potential biomedical applications. For example, the integration of superhydrophobic coatings into biomaterials may aid in controlling the protein adsorption [70], cellular interaction [88], and bacterial growth [89] that can result from body–material contact. Additionally, superhydrophobic surfaces can help enhance the manufacturing process for drug delivery technologies, for example, by serving as protein-adhesive surfaces to anchor protein-coupled hydrogel beads for UV curing [90]. 

Mosquitoes possess eyes with a highly ordered micro-nano hierarchical arrangement that resists fogging, which occurs with the accumulation of droplets with diameters of 190 nm [15]. These droplets can scatter light, causing obscured vision when they condense on eye surfaces. The mosquito compound eye consists of several ommatidia or units, which themselves consist of two parts: the dyotropic apparatus and a receptor layer of retinular cells. The dyotropic apparatus comprises a lens and four cone cells, as well as a cuticular cornea that is covered with corneal nipples that increase the amount of light passing through the dyotropic apparatus [91]. The corneal nipples are arranged in a hexagonally non-close-packed pattern on the surface of ommatidia that are arranged in a hexagonally close-packed array. This organization makes it possible for the corneal nipples to create a cushion of air in the interstitial space that increases the hydrophobicity of the surface and thereby prevents fog drops from accumulating on the eye and obstructing vision [15]. 

Liu and colleagues developed a textured metal coating that gains its superhydrophobic character by mimicking the complex anatomical structure of a mosquito’s eye [18]. As shown in Figure 6B, the mosquito eye is composed of several microscale hemispheres covered with the nanoscale corneal nipples. To construct a synthetic structure resembling the eye’s nano-framework, an aluminum alloy (6061) slab was first subjected to chemical treatments to remove imperfections. Afterward, a nanoporous layer was anodized on the surface of the aluminum alloy substrate. Tunable hydrophobic features were integrated on the surface through the addition of a self-assembly hydrophobic sol–gel, consisting of 1H,1H,2H,2H-perfluorodecyltrimethoxysilane (PFDS), ethanol, and titanium dioxide (TiO_2_) nanoparticles as a nanofiller. The TiO_2_ nanoparticles replicate the native structural function of polysaccharide chitin, a major component of the mosquito extracellular matrix. Due to its low surface energy, the chitin contributes to the eye’s hydrophobicity. PFDS has an even higher intrinsic water contact angle (WCA) than the naturally occurring chitin, and the addition of PFDS significantly increased the WCA of the fabricated surface from 150.1° (slightly superhydrophobic), as observed on an actual mosquito eye, to 168° (moderately superhydrophobic). While this coating has yet to be specifically evaluated for biomaterial uses, it has potential for medical applications, due to the biomaterial relevance of superhydrophobic characteristics in general. 

Nanostructured biodegradable coatings have also been modeled after the corneal surfaces of the *Drosophila melanogaster* fruit fly. A reverse-engineering method was used to create replicas resembling the anatomy and chemical composition of the *Drosophila* eye. Recombinant proteins and commercial waxes that respectively replicate the composition of retinin protein and corneal waxes found in a *Drosophila* eye were used as building blocks in forming nanostructured surfaces to duplicate the anatomy of the eye’s surface [78]. This protocol could be potentially modified to use mosquitoes as a model instead of fruit flies to create similar nanostructured materials that are relevant for biomaterial and medical-device industries.

### 3.3. Mosquito-Inspired Biosensor for Disease Detection

Mosquitoes use their sense of smell, or olfaction, to execute foraging, oviposition, mating, and host-seeking tasks [4]. The detection of human hosts, such as through the sensing of exhaled human breath, requires an integration of thermal, visual, and chemosensory cues, especially olfactory cues [92,93]. Ammonia, amines, carboxylic acids, lactic acid, ketones, sulfides, carbon dioxide, and 1-octen-3-ol (octenol) are among a number of volatile compounds released by humans through skin, sweat, or breath that attract mosquitoes [22,94]. The odors emanating from a host are sensed via olfactory receptors (ORs) found on the mosquito antennae, maxillary palps, and proboscis that activate olfactory sensory neurons and transmit the resulting electrical signals to the brain [4,92,93]. Female mosquitoes rely heavily on this olfaction sensing modality to locate the source of their bloodmeal from humans and other mammals [4,79]. 

Researchers have investigated the mechanisms of mosquito olfaction to devise strategies for minimizing the spread of mosquito-borne diseases that afflict humans. Here we draw focus toward research efforts to harness the chemosensing apparatus of mosquitoes in building mosquito olfaction-inspired devices for the detection of disease-specific volatile organic compounds (VOCs) [4]. Female *Aedes aegypti* mosquitoes have ORs and olfactory receptor coreceptors (Orcos) that interact with octenol molecules with particularly high sensitivity, contributing to human host attraction [95]. Manmade VOC sensors have not yet approached the level of sensitivity that *Ae. aegypti* have for octenol, which has also been reported as a biomarker candidate for diseases, including liver cancer [19,96]. Below, we describe a biohybrid-VOC sensor designed to achieve accurate detection of octenol in human breath through the incorporation of mosquito-based receptors for octenol [96].

Yamada and colleagues have integrated the *Ae. aegypti* mosquito OR and Orco (OR–Orco) into the design of a highly sensitive VOC sensor that detects gaseous octenol in human breath on the parts per billion scale. The biohybrid olfactory detector, which is depicted in Figure 6C, consists of 16 well pairs that contain a drop of medium per well and are interconnected by microchannels for VOC delivery. Wells in each pair are separated by a microporous divider that supports the formation of lipid bilayer with reconstituted OR–Orco. A superhydrophobic-coated microslit existing at the base of one compartment in each doublet enables optimal gas flow for transport of VOCs, and the flow rate can be adjusted to facilitate stirring of droplets via convection. The fluidic system detects octenol through the OR–Orcos reconstituted within the lipid bilayers, which facilitate the dissolution of octenol into liquid droplets for detection. Each well also contains an Ag/AgCl coating for the measurement of electrical signals from OR–Orcos to confirm octenol detection [19]. 

VOCs have demonstrated potential as biomarkers for the diagnosis of several other diseases, such as gastrointestinal disease [97], urinary tract infection [98], and wound infection [99]. To tune sensor functionality, it is possible to engineer OR and Orco proteins from mosquitoes or other insects to be selective for a specific VOC metabolite, and this selectivity could heavily contribute to the precision of a sensor device [19]. The customizable disease-volatile sensing device presented here may provide a new avenue for performing less invasive and time-consuming diagnostic tests for a wide range of diseases.

## 4. Conclusions

Insects are marvels of nature that have a lot to offer in terms of inspiration for developing a variety of biomaterials, treatment strategies, and medical devices. As demonstrated by the studies highlighted in this review, the anatomy and physiology of mosquitoes provide a particularly rich model for developing reproducible technologies that can be used to treat, prevent, detect, and limit disease and injury in patients. 

Mosquito-based innovations have been shown to have potential for numerous clinical applications, including medical devices, regenerative medicine, tissue engineering, therapeutics, and drug delivery. Microneedles and probes modeled after the elegant and sophisticated architecture and function of the mosquito proboscis may lead to easier and less painful medical treatments, which could increase patient compliance for medical injections. Anti-thrombotic proteins found in mosquito saliva are potential candidates for integration into biomedical implantation strategies to reduce the likelihood of device failure and adverse clinical outcomes. Multi-stimuli-responsive mosquito-derived RLPs exhibit promise as constituents of tissue scaffolds and targeted drug delivery systems. Patterned superhydrophobic surfaces have been developed based on the micro- and nanoscale anatomy of the mosquito eye. Additionally, the fine-tuned mosquito olfactory nervous system has inspired the design of sensors for diagnostic tests that can be performed with less time and invasiveness than some existing methods.

Mosquitoes possess multiple different properties that researchers are learning to harness in the development of advanced technologies for the benefit of human health. The existing and prospective mosquito-based biomedical technologies presented here are relatively new and novel. It is possible that many other mosquito features have yet to be discovered and evaluated for their potential to solve the current problems and inefficiencies in treating and diagnosing patients. The innovations presented in this review arise from early studies that have laid a foundation for the continued development of promising mosquito-inspired clinical tools in the future.

## Figures and Tables

**Table 1:** Summary of shape, material, size, and mimicking characteristics of mosquito-inspired needles (L = length, W = width, H = height, D = diameter, ID = inner diameter, OD = outer diameter, T = thickness, DP = depth, P (teeth) = pitch, CS = cross-section, ∡ = angle, * biocompatible).

Microneedle Type	Mimicked Part	Material	Dimensions	Reference
Hollow	Labrum	SU-8	D_inner_ = 100 μm, H = 1540 μm, T_wall_ = 15 μm	[37]
Hollow	Labrum	Stainless steel, 2-methacryloyloxyethyl phosphorylcholine internal coating	D_inner_ = 50 μm, D_outer_ = 90 μm, tip ∡ = 10^o^	[38]
Hollow	Labium	Titanium	D_inner_ = 25 μm, D_outer_ = 50 μm or 60 μm, L = 3.8/4 mm	[39,40,41]
Hollow	Labium	Human hair	D_inner_ ≥ 10 μm, D_outer_ = 80 μm, L = 1.1 mm	[42]
Hollow, fiber optic	Labium	Silica	*Sharp Tip:* D_inflection_ = 2 − 8 μm, D_base_ = 73 − 125 μm, L = 3 mm *Flat Tip:* D_outer_ = D_base_ = 125 − 139 μm, Taper ∡1 = 0 − 0.9°, Taper ∡2 = 3.2 − 10.7°, L = 3 mm	[43]
Hollow, jagged	Labrum; maxillae	Silicon dioxide, poly-Si coating	L = 1 mm, T_wall_ = 1.6 μm	[35]
Solid, straight, 3D sharp tips	Labrum	Silicon or polylactic acid	L = 1 mm, W = 150 μm, Tip ∡ = 18^o^	[28]
Solid, straight	Labrum	Silicon, parylene coating *	L = no limit; tip ∡ = 30^o^ or 60^o^	[31]
Solid, straight	Labrum	Silicon, parylene coating *	L = 1.0 mm, W = 60 μm, T = 100 μm	[29]
Solid, straight, biodegradable	Labrum	Polylactic acid	W = 120 − 230 µm, T = 60 − 115 µm, Tip ∡ = 15 − 75°	[30]
Solid, jagged	Maxillae	Silicon, parylene coating *	L = 1.0 mm, W = 60 μm, T = 100 μm, P_teeth_ = 10 μm	[29]
Solid, jagged	Maxillae	Stainless steel	L = 2.2 mm, W = 70 µm, Tip ∡ = 15^o^, P_teeth_ = 20 µm, DP_teeth_ = 7 µm	[33]
Solid, jagged or harpoon, long, 3D sharp tips	Maxillae	Silicon, parylene coating *	L = no limit; Tip ∡ = 30^o^ or 60^o^	[31]
Solid, jagged, biodegradable	Maxillae	Polylactic acid	Tip ∡ = 30^o^	[30]
Solid, hooked, biodegradable	Maxillae	Polylactic acid	Tip ∡ = 30^o^	[30]
Combination, 2-part (alternatively moving halves), jagged, hollow, holes in walls	Labrum; maxillae	IP-S	D_inner_ = 50 μm, D_outer_ = 100 μm, L = 1 − 2 mm	[34]
Combination, 3 needles (1 central straight, 2 outer harpoon-like jagged), *fixed* (with bridges) or *free* (with no bridges)	Labrum; maxillae	Silicon, parylene coating *	*Central needle:* L = 1.0 mm, T = 100 μm, W = 30 μm *Outer needles*: L = 1.0 mm, T = 100 μm, W = 15 μm	[29]
Combination, 3 needles (1 central straight and hollow, 2 outer jagged)	Labrum; maxillae	IP-S; IP-Dip^TM^	*Central needle* (cone shape): D_base_ = 30 μm, H_base_ = 100 μm, Tip D_inner_ = 20 μm, Tip D_outer_ = 30 μm *Outer needle* (solid cylinder): D_inner_ = 40 μm, D_outer_ = 50 μm; 14 graded serrated projections (W = 0.6 − 2.0 μm, H= 0.8 − 6.0 μm, L = 1.0 -8.0 μm) *All needles:* L_total_ = 2 mm, Inter-needle gap = 10 μm	[21]

**Table 2:** Five biomolecules derived from mosquito saliva that are summarized based on their biological mechanisms of action and mosquito species characteristics.

Biomolecule	Mosquito Type	Vector-Borne Human Disease	Mechanism of Action	Reference
Anopheline Antiplatelet Protein (AAPP)	*Anopheles stephensi*	Malaria, Lymphatic Filariasis	Binds collagen; inhibits interaction with glycoprotein VI and integrin 21	[2,10]
Aegyptin	*Aedes aegypti*	Yellow Fever, Chikungunya, Zika Fever, Dengue Fever	Binds to collagen preventing its interaction with von Willebrand factor, integrin a2b1, and glycoprotein VI; inhibits factor Xa	[6,7,57]
CCP Protein	*Culex pipiens pallens*	Japanese Encephalitis, Lymphatic Filariasis West Nile Virus	Inhibits enzymatic activity of thrombin and factor Xa; may inhibit interaction between coagulation factors and platelet receptors	[22,8]
Hamadarin	*Anopheles stephensi*	Malaria, Lymphatic Filariasis	Inhibits activation of plasma contact system by binding to factor XII and high-molecular-weight kininogen	[2,11]
Heparin	*Aedes togoi*	Japanese Encephalitis, Filariasis, Yellow Fever	Inhibits thrombin and factor Xa by activating antithrombin	[9]

## Data Availability

Not applicable.
